# Performance of an Integrated Membrane Process with Electrochemical Pre-Treatment on Poultry Slaughterhouse Wastewater Purification

**DOI:** 10.3390/membranes10100256

**Published:** 2020-09-24

**Authors:** Kulyash Meiramkulova, Davud Devrishov, Mikhail Zhumagulov, Sholpan Arystanova, Zhaskhaiyr Karagoishin, Saida Marzanova, Aliya Kydyrbekova, Timoth Mkilima, Jianxin Li

**Affiliations:** 1 Department of Environmental Engineering and Management, Faculty of Natural Sciences, L.N. Gumilyov Eurasian National University, Satpayev Street 2, Nur-Sultan 010000, Kazakhstan; kuleke@gmail.com (K.M.); sholpan1607@mail.ru (S.A.); aliyafromkz@gmail.com (A.K.); 2 Department of Immunology and Biotechnology, Moscow State Academy of Veterinary Medicine and Biotechnology, 23 Scryabin str, Moscow 109472, Russia; davud@mgavm.ru (D.D.); s.marzanova@mail.ru (S.M.); 3 Department of Thermal Engineering, L.N. Gumilyov Eurasian National University, Satpayev Street 2, Nur-Sultan 010000, Kazakhstan; mikelike2000@yandex.kz; 4 Department of Hunting and Fishering, Faculty of Veterinary Sciences and Animal Husbandry, Saken Seifullin Kazakh Agricultural Technical University, Satpayev Street 2, Nur-Sultan 010000, Kazakhstan; k.zhashayir@mail.ru; 5 Department of Civil Engineering, Faculty of Architecture and Construction, L.N. Gumilyov Eurasian National University, Satpayev Street 2, Nur-Sultan 010000, Kazakhstan; 6 State Key Laboratory of Separation Membranes and Membrane Processes, National Center for International Joint Research on Separation Membranes, School of Materials Science and Engineering, Tiangong University, Tianjin 300387, China; jxli@tiangong.edu.cn

**Keywords:** poultry wastewater treatment, electrolysis, membrane filtration, cake formation, water quality

## Abstract

Industrial activities produce a variety of pollutants that may not be easily treated using centralized wastewater treatment systems based on a single treatment unit. The variability of the pollutants brings the importance of industrial-specific integrated wastewater treatment plants such as integrated membrane filtration systems. However, the performance of a membrane filtration process can be highly affected by the presence of high amounts of suspended particles in the raw wastewater. Therefore, proper selection of a pre-treatment unit prior to a membrane filtration wastewater treatment system is a key aspect of its performance. This study investigated the performance of an integrated membrane filtration treatment system connected to an electrochemical process (pre-treatment) on the purification of a poultry slaughterhouse wastewater toward achieving a high-quality effluent. The industrial-scale treatment plant installed at the Izhevsk Production Corporative (PC) poultry farm in Kazakhstan is composed of an electrochemical, ultrafiltration (UF), and reverse osmosis (RO) as the main treatment units. From the analysis results, the electrochemical pre-treatment unit was observed to be highly effective for the removal of some physicochemical parameters such as turbidity, color, total suspended solids, total iron, aluminum, chemical oxygen demand, and biochemical oxygen demand; with removal efficiency ranging from 71 to 85%. The low removal efficiency of the pre-treatment system was also observed from free and total chlorine, nitrites, nitrates, phosphates, and ammonium nitrogen; with removal efficiency ranging from 4 to 45%. While in general, the overall treatment train was observed to be highly efficient for some physicochemical parameters such as turbidity, color, total suspended solids, as well as chemical and biochemical oxygen demand; maintaining almost 100% removal efficiency throughout the study period. Also, the high removal efficiency of the electrochemical pre-treatment processes led to a relatively low rate of cake formation on the membrane filters.

## 1. Introduction

The affordability of poultry products makes the poultry industry one of the highly growing industrial sectors in the world. However, the whole process from growing birds to meat production is water-demanding [1]. It is approximated that broilers consume 1.6 to 2.0 times as much water as feed on a weight basis; the high water consumption is characterized by the fact that water is a critical nutrient in bird metabolism and nutrition [2]. Unfortunately, the more water is consumed, the more wastewater is generated; the phenomenon makes the poultry industrial sector one of the high wastewater generators [3]. The challenge of poultry production wastewater is not only on the quantity of the wastewater but also the high pollution strength of the generated wastewater, characterized by the richness in organic matter, nitrogen, and phosphorus [4]. Because of the potential risks posed by such highly polluted wastewater to human health and the environment in general, the treatment of poultry production wastewater before discharge or reuse has been of major concern [5]. Moreover, the technical and economic feasibility of a poultry production wastewater treatment is highly dependent on the technology used [6,7,8]; which also includes the scale at which the particular treatment system is operating [9]. Therefore, having a clear knowledge of a treatment technology ability before its application for poultry wastewater treatment is of significant necessity; which in turn brings the importance of conducting research.

Different technologies have been employed for poultry slaughterhouse wastewater treatment, with electrochemical (EC) methods [10], aerobic and anaerobic systems [11,12], as well as submerged fibers [13], as examples. However, the variability of pollutants in the poultry slaughterhouse wastewater makes it difficult to achieve high-quality removal efficiency using a single treatment unit. For example, the main advantage of the biological treatment processes is the fact that they are capable of operating under natural conditions. However, slow treatment processes, high space requirements, and sludge generation [14] are among the drawbacks of the biological treatment processes. Moreover, chemical-based wastewater treatment systems, including electrochemical processes are referred to as robust, space-friendly, with high flexibility to fluctuating wastewater characteristics [15]. However, with chemical-based technologies, there is a high risk of by-products formation [16]. Therefore, the integration of different treatment technologies to form a composite treatment system such as a combination of EC and integrated membrane filtration (IMF) systems offers a more efficient treatment approach. In the recent past, integrated treatment technologies for poultry slaughterhouse wastewater have also been increasingly gaining interest [17,18].

The EC treatment technologies utilize electrical energy as the driving force for chemical reactions to occur. Water is decomposed to hydrogen and oxygen by passing a current through it in the presence of electrolytes [19]. In general, the EC treatment process offers two main possibilities for the removal of pollutants with the principal mechanism being oxidation of pollutants to carbon dioxide (CO_2_) and water, which is also known as EC combustion or mineralization, as well as to biodegradable products. The first removal possibility is based on the direct anodic oxidation, but this process leads to very poor decontamination. The second removal option is based on the chemical reaction with electrogenerated species from water at the anode; which include physisorbed hydroxyl radical (OH) and chemisorbed oxygen in the lattice of a metal oxide (MO) anode, of which the action of these oxidizing agents leads to total and partial removal of pollutants, respectively [20].

Membrane filtration (MF) processes are pressure-driven separation mechanisms using membrane filters for both mechanical and chemical sieving of particles as well as macromolecules [21]; the pressure needed to press water through a membrane is known as trans membrane pressure (TMP) [22]. MF systems are preferred unit operations in industrial applications because of their mild operating conditions including better effluent quality, smaller footprint, decreased sludge production, and convenient automated control [23]. However, the performance of a membrane stack drops over time because of cake formation on the membrane surface [24,25]. Cake formation in almost all membrane processes is normally caused by precipitation and deposition of molecules or particulates on the membrane surface causing pore blocking, hence resulting in cake formation [26]. The consequences of membrane cake formation are, increased membrane separation resistances reduced productivity, as well as altered membrane selectivity leading to higher costs of energy, operation, and maintenance [27]. This affects the separation factor for targeted species in the feed, with a consequence of unstable product quality and poor recovery [28].

The major factors that influence membrane cake formation in wastewater treatment can be categorized into three groups, which are: membrane properties including material from which the membrane is made, characteristics of the wastewater as well as the performance of the pre-treatment unit [28]. However, the extent to which the cake formation phenomenon will have an effect on the MF process to a great extent is highly dependent on the combination of the characteristics of wastewater to be treated and the type of pre-treatment technology used [29]. Therefore, it is of great importance to understand the behavior of a pre-treatment technology in relation to the characteristics of the wastewater to be treated to reduce membrane cake formation as well as increasing the contaminant removal efficiency [30]. In general, although the EC and IMF treatment combination has some applications in other fields of wastewater, the process is not famous in the field of poultry slaughterhouse wastewater, especially on an industrial scale. The information about the technical and economic feasibility of the process at an industrial scale is still scarce. Also, the arrangement of aluminum (anode) and graphite (cathode) for poultry slaughterhouse wastewater treatment presents a piece of valuable information in the field.

In this study, the performance of integrated membrane filtration (IMF) system connected to EC treatment system as a pre-treatment unit on the purification of a poultry slaughterhouse wastewater toward achieving a high-quality effluent is investigated. The industrial-scale treatment plant installed at the Izhevsk PC poultry farm in Kazakhstan is composed of an EC oxidation process, ultrafiltration (UF), and reverse osmosis (RO). The general performance of the entire treatment plant with time is investigated. Also, the effluent quantity and cake formation on the membrane filters were monitored over time. The cake thickness estimation was achieved by measuring the thickness of the residue deposited upon the filter for six months. As high-quality effluent with a recycling potential is required from the treatment plant; in some cases, the Canadian standards for drinking water quality, aquatic life protection as well as the irrigation water are referred. The Canadian water quality standards were selected because of the wide range of the defined standards. Also, the World Health Organization (WHO) standards for drinking water quality as well as the standards set by the government of Kazakhstan were used for the percent compliance analysis.

## 2. Materials and Methods 

### 2.1. Case Study and Water Samples

The water samples were collected from the Izhevsk PC poultry slaughterhouse located in Izhevsk village, Arshalinsky district, in Akmola region of the Republic of Kazakhstan, about 70 km from the capital city Nur-Sultan (51° 10’ North latitude and 71° 26’ East longitude). Samples were collected as grab samples before treatment (raw wastewater), EC process effluent as well as after the IMF process using 5L plastic bottles, which were thoroughly rinsed with deionized water before use. Replicate samples were also collected and analyzed for data quality control. All samples were preserved at 4 °C before analysis. The analysis of the samples was done at the L.N. Gumilyov Eurasian National University (ENU) Water and Environmental Management lab in Nur-Sultan, Kazakhstan.

**Figure 1:**
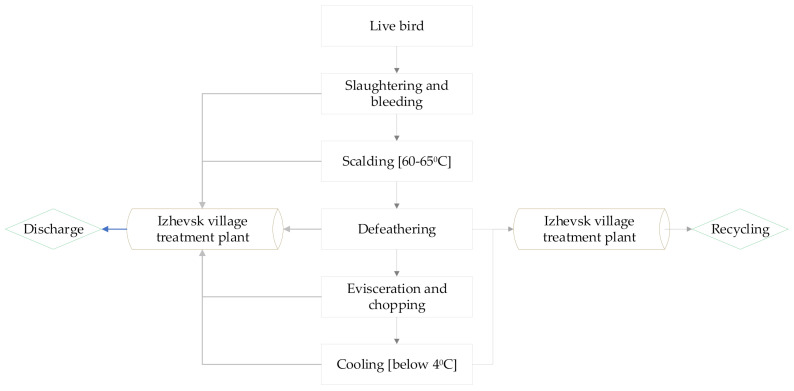
Production and wastewater flowchart.

The production processes of the slaughterhouse generate wastewater from the live bird processing to the cooling section; with defeathering, evisceration, and cooling processes being the main sources of wastewater generation in the slaughterhouse (Figure 1). The poultry farm intends to recover some of the wastewater generated from the defeathering and cooling sections of the slaughterhouse which account for the highest amount of the wastewater among the sources. The recovered wastewater after purification is used for cooling purposes; while the other amount of wastewater is discharged to the sewerage system.

Table 1 presents the general characteristics of the raw wastewater in terms of minimum concentration values (Min), maximum concentration (Max), arithmetic mean or average (AM), as well as standard deviation (SD) from 17 water quality parameters (pH Turbidity, Color, total suspended solids (TSS), free chlorine, total chlorine, nitrites, nitrates, phosphates, ammonium, total iron, aluminum, chemical oxygen demand (COD), biochemical oxygen demand (BOD), chromium, nickel, and manganese).

### 2.2. Treatment Plant Setup and Characteristics

The Izhevsk PC wastewater treatment plant is an integrated wastewater treatment system consisting of several treatment units connected in series. The system is characterized by a semi-batch process as it relies heavily on the production activities in the slaughterhouse. Moreover, clean water (treated effluent) from the treatment plant is approximately 60% of the raw wastewater fed to the treatment system. The general characteristics of the treatment plant are summarized in Table 2.

#### 2.2.1. Pre-treatment Processes

The principal objective of the pre-treatment processes was to ensure that the feed water to the IMF system is compatible with the membranes. Pre-treatment was introduced as an important aspect before the IMF treatment processes to specifically increase the efficiency and life expectancy of the membrane elements by minimizing fouling, scaling as well as degradation of the membranes.

Raw wastewater from the slaughterhouse production processes is first stored in a receiving tank which also acts as the primary sedimentation and cooling unit with a capacity of 1 m^3^. Mesh filter has been installed above the receiving tank with a mesh size of 1.2mm x 12mm. An ultrasonic water metering unit is provided in front of the receiving tank. The receiving tank is equipped with three-level sensors; the upper level, the lower level sensor, and the sensor to protect the pump from an empty move. The drain pump is switched on when water is filled up to the upper level. The treatment process is followed by feather catching, fat catching, as well as coarse mechanical filtration (Table S1 in Supplementary Materials). The wastewater is fed to the coarse filtration process using a rotary pump, with a mesh size of 1 mm; where all large impurities are left on the grid. The sludge containing large impurities is manually discharged into the sewerage system.

The EC treatment system is the main pre-treatment unit within the plant prior to the IMF treatment processes. The length of the EC system reactor is 1040 mm (1.04 m), width is 790 mm (0.79 m), and height is 1540 mm (1.54 m). A combination of graphite (cathode) and aluminum (anode) electrodes is used with a power supply of 380 volts. The level sensor of the float type serves as a control signal to turn on the power unit of the electrolyzer; which is installed directly inside the EC chamber. The state of the electrolyzer is regularly monitored; while the on and off operations are carried out both in manual and in automatic modes. The operating current and voltage of the EC treatment unit are also manually controlled and adjusted.

#### 2.2.2. IMF Treatment Processes

The IMF treatment system is composed of ultrafiltration and reverse osmosis. The integrated system consists of one module of ultrafiltration and one module of reverse osmosis. The ultrafiltration process is carried out by passing the pre-treated water through a cartridge-type filter for an ultrafine cleaning. The pump NS 1 creates the necessary pressure for the flow of water through the filter material with a pore size of 0.02 μm or 1760 kDa [Aquafor LLP, Moscow, Russia]. Table S2 in Supplementary Materials presents the general technical specifications of the ultrafiltration unit.

The reverse osmosis treatment system is installed in a fiberglass housing, connected by a piping system with a capacity of 1 m^3^/h of pure water. The membrane filter pore size is 0.0001 μm (0.22 Da) [Toray brand, TM720D-400] manufactured by the Japan Toray Industries Inc. The EBARA EVMS 3 16F5/1.5 pump (produced by Japan Ebara Corporation, Tokyo) is used for creating the required pressure to force water through the membrane. During this process, the flow is divided into purified water (permeate) and concentrate, which is subsequently discharged into the sewerage system, or recycled for cooling purposes in the industry. Other general technical specifications are summarized in Table S3 in Supplementary Materials.

The hydraulic control section of the reverse osmosis consists of:

- A manometer for measuring the pressure difference at the inlet to and from the cartridge filter.
- Pressure gauge for measuring pressure at the inlet to the filter and reverse osmosis and at the outlet of the permeate line.
- Pressure diminution equipment on the suction line to protect the unit from reduced pressure.
- The pressure gauge on the pressure line of the booster pump.

### 2.3. Experimental Setup

In this study, samples were collected in three different sources to investigate the wastewater characteristics, the performance of the pre-treatment unit, and the general performance of the treatment system. For the experimental purposes, the connection to the IMF from the EC treatment system was modified and equipped with an outlet mechanism for easy sampling. The aluminium electrode was fixed as anode and the graphite electrode was fixed as a cathode unit to investigate their feasibility for poultry slaughterhouse wastewater treatment at an industrial-scale. Multimeters were also installed for the operating current and voltage monitoring. To avoid variability in terms of treatment efficiency and cake formation, new membrane filters were installed at the beginning of the study and were not changed throughout the study period. The manual method (using a ruler on multiple locations) was used to determine the cake thickness layer. However, not less than 30 measurements were to be carried out per session of the study to obtain optimum results; this is due to variations in cake thickness on the membrane surface as well as taking into account the possible errors caused by the partly washed away filter cake and deformation caused by the ruler.

### 2.4. Analytical Methods

The research team ensured that the collected samples are analyzed within the same day of collection. Chemical parameters were mainly measured using the combination of spectrophotometer (Hach DR3900, HACH/LANGE, Berlin, Germany), colorimeter (Hach DR900, HACH/LANGE, Berlin, Germany), with standard reagents as well as the test kits provided by Hach Company, [31]. The Standard Operating Procedure for GLNPO Turbidity (The U.S. Environmental Protection Agency Great Lakes National Program Office, Washington, D.C.) was used for the analysis of turbidity [32], and the American Public Health Association (APHA) 4500-Nor, Washington, D.C., United States, was used for the analysis of total phosphorous, while lab pH-meter (Hach Co, HACH/LANGE, Berlin, Germany) was used for pH as well as the ultraviolet-visible (UV-V) spectrophotometer (PE-5400UV) pr-in ECOCHEMICAL, St. Petersburg, Russia was used for color measurements. The TSS in the samples was determined using Hach TSS portable hand-held turbidity meter. In general, the analyses of all the studied samples were accomplished following the recommendations in the APHA Standard Methods for the Examination of Water and Wastewater [33].

### 2.5. Statistical Methods

Minimum and maximum concentration values were computed using the Microsoft Excel 2019 in-built functions. Also, arithmetic mean, standard deviation, and percent removal efficiencies were computed from the data series of each of the studied water quality parameters. Correlation analysis was performed for some of the physicochemical parameters to evaluate the strength of the relationship among the selected parameters. This means a high correlation indicates that two or more variables have a strong relationship with each other. For a case of weak correlation means that the variables are hardly related. The interpretation of the correlation coefficients used in this study is summarized in Table 3

Box and whisker plots were used to evaluate the data distribution (skewness) as well as identifying whether there were potential unusual observations (outliers) in the datasets. Also, they were used to compare results before treatment, after EC purification, as well as after the IMF treatment processes.

Treatment efficiencies from raw wastewater and the purified water analysis results were calculated as summarized in Equation (1).

(1) $$T_{e}\left(\%\right)=\left(\frac{C_{b}-C_{a}}{C_{b}}\right)\times 100$$

where;

$T_{e}$, treatment efficiency,
$C_{b}$, concentration before treatment,
$C_{a}$, concentration after treatment.

Analysis of the final effluent compliance with different drinking water quality guidelines was done, mainly based on the guidelines set by the WHO, except for TSS, chlorine total, phosphates, and ammonium where Kazakhstan guidelines were adopted. The drinking water quality standards were selected because the treated effluent has to be recycled for cooling purposes within the slaughterhouse. Equation (2) gives a summary of the approach used for compliance computation.

(2) $$C_{p}\left(\%\right)=\left(\frac{S_{i}-C_{i}}{S_{i}}\right)\times 100$$

where;

$C_{p}$, percent compliance,
$S_{i},$ the recommended standard for an i^th^ parameter,
$C_{i},$ the concentration of the i^th^ parameter.

Daily power consumption for each treatment unit was investigated over time, from this data the power consumption for each m^3^ of treated effluent was estimated and used for the computation of the operating cost.

In general, the statistical analysis of the results presented in this study was achieved using Microsoft Excel 2019.

## 3. Results and Discussion

### 3.1. Physicochemical Parameters

The collected samples were successfully analyzed for physical and chemical parameters. A total of 17 parameters (pH, turbidity, color, TSS, free chlorine, total chlorine, nitrites, nitrates, phosphates, ammonium, total iron, aluminum, COD, BOD, chromium, nickel, manganese) were investigated.

#### Turbidity, TSS, Color, BOD, and COD

Table 4 presents the results of the physicochemical water quality parameters analyzed at the ENU Environmental and Water Management lab. Before treatment; the minimum, maximum, and average concentration values for turbidity were 68.70, 647.00, and 259.62 FAU, respectively. However, after the EC treatment process, 1.08, 114.00, and 70.32 FAU values for minimum, maximum and average concentrations were achieved, respectively, which is equivalent to 98.43%, 82.38%, and 72.91% change of the minimum, maximum, and average concentrations, respectively. According to the WHO drinking water quality standards [34], the turbidity should be kept below 5 FAU. In this case, from the average turbidity concentration, the IMF treatment system is of high potential to further reduce the concentration of turbidity to acceptable levels.

TSS removal in feed water prior to a membrane filtration treatment process is important to improve membrane flux [35]; especially for highly polluted wastewater such as the one generated from the poultry slaughterhouse activities. In the raw wastewater, minimum, maximum, and average recorded TSS concentrations were 116.00, 1068.00, and 452.67 mg/L, respectively. After the EC treatment process, 13.00, 198.00, and 128.25 mg/L were achieved as minimum, maximum, and average concentrations, respectively; achieving an average removal efficiency of more than 71%.

High BOD concentrations were also observed in the raw wastewater; with 1220, 1409, and 1321.33 mg/L being minimum, maximum, and average concentrations, respectively. Also, the recorded COD concentrations from the raw wastewater ranged from 1274 to 7401 mg/L, with 3527.17 mg/L being the average concentration. The high concentrations of BOD and COD can be linked to the fact that the poultry slaughterhouse activities are associated with a high generation of blood and complex mixture of fats, proteins, and fibers, which contribute to the increasing the organic matter [36]. After the EC treatment, the average removal efficiencies of more than 85% and 78% were achieved for BOD and COD, respectively. In the literature, BOD and COD removal efficiencies from different wastewater sources were observed to be ranging from 73 to 85% using EC treatment methods [37,38,39].

The concentrations of free and total chlorine were observed to be low in the raw wastewater; 0, 0.9, and 0.23 mg/L were recorded as the minimum, maximum, and average concentration values in the raw wastewater for free chlorine, respectively. While 0.03, 1.04, and 0.28 mg/L were recorded as a minimum, maximum, and average concentrations for total chlorine, respectively. The low chlorine concentration in the raw wastewater is due to the fact that the poultry farm intend to achieve a chlorine-free production system to improve the general quality of the products as well as environmental conservation in general. After the EC treatment process, 0, 0.22, and 0.15 mg/L were recorded as the minimum, maximum, and average concentrations of free chlorine, respectively; with an average removal efficiency of 34.78%. Also, 0, 0.24, and 0.18 mg/L were recorded as minimum, maximum, and average concentration of total chlorine after the EC treatment; with an average removal efficiency of 35.71%.

Nitrogen and phosphorus are referred to as nutrients; nitrogen, in the form of nitrate, nitrite, as well as ammonium, is an essential nutrient needed for plant growth. But, when the water bodies receive a relatively high amount of nutrients, they are likely to be polluted by excessive growth of algae endangering fish and other aquatic life. Moreover, according to studies, some forms of algae such as blue-green may produce toxins with possibilities of being harmful if ingested by humans and animals [40]. The EC pre-treatment system faced a significant challenge in nutrients removal. Average removal efficiencies of 33.01%, 12.5%, 34%, as well as 4.19% were achieved for nitrate, nitrite, ammonium, and phosphates, respectively. In the literature, it has been observed that the efficiency of ammonium removal in wastewater can be highly affected by the type of electrode material used, HRT, pH, and the current density applied [41,42]. In this study, electrode material, HRT, pH, and current density were not adjusted to investigate their potential influence on ammonium nitrogen removal. However, Umesh et al. [43] investigated the removal of ammonium nitrogen using the EC method with platinum-coated titanium; during the seven-hour experiment, as low as 10% removal efficiency was achieved following some adjustments to the current density. Also, Hanspeter et al. [44] studied direct electrochemical oxidation of ammonia on graphite and observed that a pH value greater than 9 is required for effective removal of ammonia. However, in this study, the pH values in the raw wastewater ranged from 6.80 to 7.40 with 7.13 being an average value. Similar factors are highly linked to the challenges in the removal of phosphates, nitrates as well as nitrites. Therefore, the selection and application of an EC pre-treatment method for nutrients removal have to properly observe all these parameters.

Chromium, nickel, and manganese are among the potentially toxic elements (PTEs) released from poultry production activities [45]. The Canadian water quality guideline for chromium in drinking water is 0.1 mg/L [46] and the protection of freshwater life is 0.005 mg/L [47]. A water quality guideline for hexavalent chromium of 0.008 mg/L in irrigation water is recommended for the protection of agricultural crop species [48]. The average chromium concentration in the raw wastewater is about 11.9 times higher than the recommended guideline for drinking water, 238 times higher for protection of aquatic life, as well as 148.75 times higher than the guideline for protection of agricultural crop species. After the EC treatment process, an average concentration of 0.72 mg/L was achieved, which is equivalent to 39.5% removal efficiency.

High levels of nickel were observed in the raw wastewater; with 1.96, 21.96, 7.53 mg/L as the minimum, maximum, and average concentrations, respectively. The potential sources of nickel in the poultry wastewater can be leaching from metals, chicken feeds, as well as offal products especially the liver [49]. The EC pre-treatment unit achieved nickel removal efficiency of approximately 52.06%. Nickel is considered to be toxic when exceeding 0.2 mg/L, leading to dermatitis, nausea, chronic asthma, coughing as well as a human carcinogen [50].

Manganese concentrations in the raw wastewater ranged from 0.29 to 0.95 mg/L with 0.72 mg/L being the average concentration. According to the Canadian guidelines, the maximum acceptable concentration (MAC) for total manganese in drinking water is 0.1 mg/L, while the aesthetic objective (AO) for total manganese in drinking water is 0.02 mg/L [51]. The average manganese concentration in the raw wastewater is approximately 7.2 times (720%) MAC according to the Canadian guidelines. After the EC treatment process, an average concentration of 0.21 mg/L was achieved for manganese; which is still 2.1 times (210%) higher than MAC according to the Canadian guidelines. However, as a pre-treatment unit, the EC process achieved a 70.8% average removal of manganese.

Moreover, lowering the concentrations of iron and aluminum before a membrane filtration treatment process is crucial because of their potential of reacting with antiscalant components to form colloidal foulants [52,53]. The average concentration of total iron in the raw wastewater was 0.90 mg/L; after the EC treatment process, the average concentration of 0.17 mg/L was achieved, which is equivalent to 81.11% removal efficiency. Also, the average concentration of aluminum in raw wastewater was 0.78 mg/L; however, after the EC treatment, an average concentration of 0.22 mg/L was achieved, which is equivalent to 71.8% removal efficiency. The high removal efficiency of the EC pre-treatment unit on total iron and aluminum plays an important role in the reduction of scaling of mineral salts onto the membrane surface as well as maintaining the design flux.

Apart from the samples collected after the EC pre-treatment processes, also samples were collected from the IMF treated effluent. From Table 5, it can be observed that after the IMF treatment process, 0 mg/L was achieved as the minimum recorded concentrations for turbidity, TSS, free and total chlorine, nitrites, ammonium, chromium, and nickel. From the concentrations of treated effluent, 0 mg/L is equivalent to 100% removal efficiency.

Based on the SD results, it can be observed that the treatment plant was able to maintain high removal consistency over time. 

From the average concentrations, 70.32 FAU of turbidity was achieved as the EC treatment process effluent, while, 0.32 FAU of turbidity was achieved as average concentration after the IMF treatment, which is equivalent to a 99.5% change from the EC effluent to the IMF effluent. Following a similar approach, 98.9% change was achieved from color as well as 99%, 98.9%, 98% from TSS, COD, and BOD, respectively.

The lowest percent change is observed from nickel and manganese with 61.8% and 42.9% respectively. The phenomenon is also reflected in the total removal efficiency of nickel and manganese, presenting the lowest removal efficiency among the studied water quality parameters.

Table 6 reveals further that, the EC pre-treatment processes had an impressive performance on the removal of turbidity, color TSS, total iron, aluminum COD, BOD, as well as manganese. However, EC low removal efficiency can be observed for parameters such as free and total chlorine, nitrites, nitrates, phosphates and total phosphorus, and ammonium nitrogen, with removal efficiency ranging from 4 to 45%.

In this study, seven parameters (turbidity, color, TSS, nitrates, phosphates, ammonium, and COD) were selected as case studies for correlation analysis. From Table S4 in Supplementary Materials, it can be seen that the correlation coefficient between turbidity and color is 0.999834, which falls under the “very strong” correlation category. In literature, turbidity and color are observed to be linearly correlated [54], of which turbidity can be created by the particulate matter resulting from particles too large to be in true solution and yet small enough to remain suspended against the force of gravity, imparting color to water [55]. A very strong correlation can also be observed between COD and other parameters such as turbidity, color TSS, nitrate, and phosphates. COD is a critical parameter of determining water quality status which can define the degree of contamination in water. Therefore the strong correlation can be linked to the fact that COD covers the total amount of oxygen consumed in water to chemically oxidize organic contaminants to inorganic end products [56].

**Figure 2:**
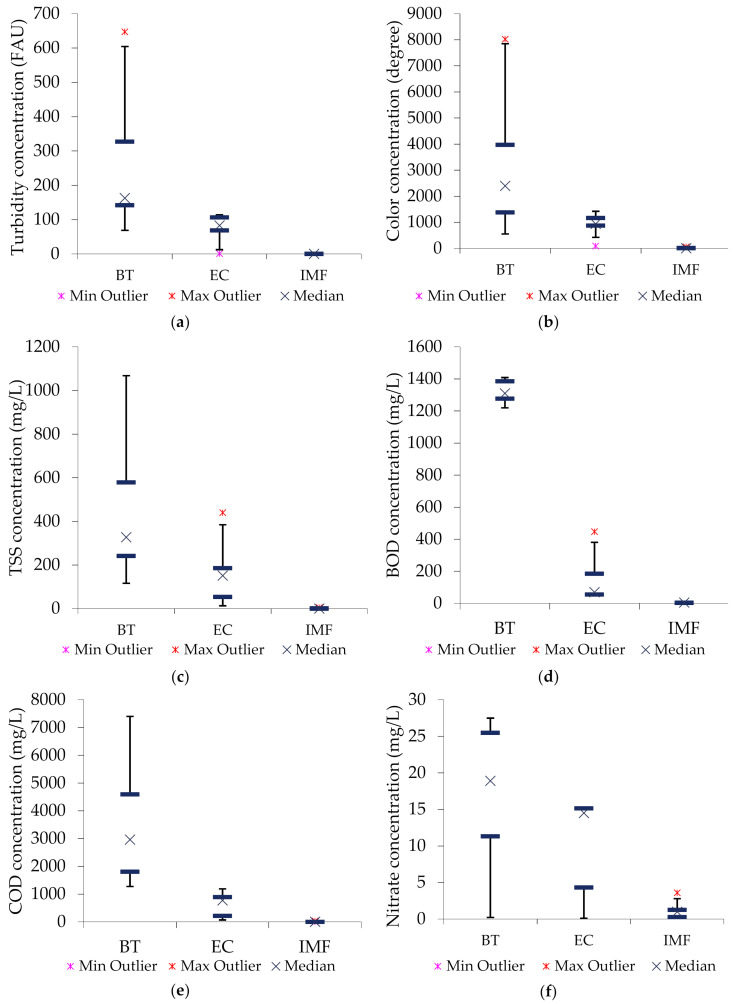
Box and Whisker plots; (**a**) turbidity, (**b**) color, (**c**) TSS, (**d**) BOD, (**e**) COD, (**f**) nitrate.

Figure 2 shows the general performance of the integrated treatment system by comparing the analysis results before treatment (BT), after electrolysis (EC), and after the IMF treatment processes. The error bars illustrate the range of the studied data, the lower quartile, median, upper quartile, and outliers are also presented. It can be observed that the purification performance increases as more treatment units are applied in the system. The concentrations of BOD in raw wastewater ranged from 1200 to 1400 mg/L with high consistency (no outliers) (Figure 2d). The highest purification performance can be seen from the IMF effluent achieving almost 0 mg/L of turbidity, BOD, COD, and TSS as well as 0 degrees of color. Figure 2f reveals further that, the pretreatment processes had a lower influence on nitrate removal which is also reflected in the final effluent. The use of the graphite electrode as a cathode has also shown to be a challenge for nitrate removal in some other studies; the phenomenon is highly linked to the low removal rate and nitrogen selectivity [57,58,59].

In general, from the Box and Whisker plots, it can be seen that the concentration of the released pollutants from the poultry slaughterhouse activities is of high fluctuation, which means a pre-treatment unit prior to the membrane filtration treatment process has to be carefully selected to withstand the highly fluctuating wastewater characteristics. The errors can also be highly linked to the relatively high fluctuation of the wastewater characteristics affected by the general complex production procedures in the poultry farm.

### 3.2. General Treatment Efficiency over Time

**Figure 3:**
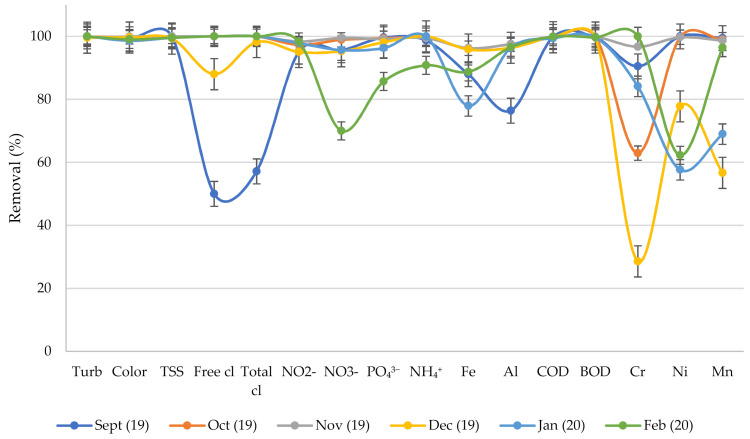
Removal efficiency over time (September-2019 to February-2020); Turb = turbidity, cl = chlorine, NO_2_^−^ = nitrite, NO_3_^−^ = nitrate, PO_4_^3−^ = phosphates, NH_4_^+^ = ammonium, Fe = iron, Al = aluminum, Cr = chromium, Ni = nickel, Mn = manganese.

Figure 3 reveals further that the integrated treatment plant is highly efficient for some physicochemical parameters such as turbidity, color, TSS, COD, and BOD. It can be observed that the treatment plant was able to maintain almost 100% removal efficiency for turbidity, color, TSS, COD, and BOD for more than six months. High removal consistency is also observed from nitrite with more than 90% removal efficiency within the selected study period. Parameters such as nitrates, phosphates, and ammonium are also observed to be associated with high removal efficiency except for February 2020. The low removal efficiencies for some months can be highly linked to the fluctuations in the influent wastewater characteristics due to the complex production processes in the poultry farm. In the literature, fluctuation in the influent quality has been observed to be among the generic problems that can affect the performance of small wastewater treatment plants [60].

Table 7 provides a summary of the percent compliance of some physicochemical parameters with drinking water quality standards. It can be observed from the average concentrations of the final effluent that most of the parameters were within the recommended standards for drinking water quality, except for color and chromium. Although general removal efficiencies of 99.72% and 94.12% from color and chromium, respectively were achieved in the final treated effluent, the concentrations of color and chromium in the final effluent were not sufficient enough to comply with the recommended limit set by the WHO. The negative values in Table 7 indicate that the particular average concentration exceeded the recommended limit.

### 3.3. Filtrate and Operating Cost Analysis

**Figure 4:**
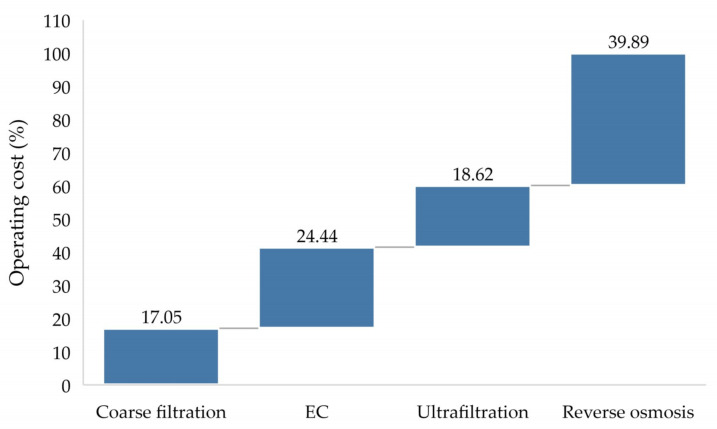
Operating cost

The estimated cost is mainly based on power consumption per cubic meter of treated water. The energy consumption for each cubic meter of the coarse filtration was approximately 0.513 kWh, electrolysis 0.7352 kWh, ultrafiltration 0.56 kWh, and reverse osmosis 1.2 kWh. Operating cost for each cubic meter, 0.016 $USD for coarse filtration, electrolysis 0.023 $USD, ultrafiltration 0.018 $USD, and 0.038 $USD for reverse osmosis. The pre-treatment units prior to IMF make up a total of approximately 41.49% of the operating cost for the entire treatment plat. From Figure 4, it can be observed that the operating cost for reverse osmosis is around twice as much as the ultrafiltration unit. With the fact that the estimated operating cost was based on power consumption, the difference in operating cost between the ultrafiltration and reverse osmosis is also highly linked to the difference in power consumption between the two units. While the ultrafiltration is just a filter unit, working under relatively low pressure, leading to low energy consumption [61], the reverse osmosis is a molecules-based separation system in which sufficient pressure is applied against feedwater to force it through the semipermeable membrane allowing only water to pass while rejecting other compounds [62]. The reverse osmosis process requires a relatively higher pressure compared to the ultrafiltration with increased requirements to pumps, more complex connections, as well as higher energy consumption [63].

From the monitored filtrate quantity, it was observed that the IMF system was able to maintain almost the same filtrate quantity for more than six months; with an average of 0.75 m^3^ per day of purified water (60%) out of 1.25 m^3^ per day of the total influent wastewater. Moreover, the ultrafiltration treatment process was observed with an average cake thickness of 0.043 m over six months; equivalent to 2.4 × 10^−4^ m per day. Also, 0.016 m six month of cake thickness was observed from the reverse osmosis; equivalent to 8.9 × 10^−5^ m per day. The relatively low rate of cake formation can be highly linked to the fact that the EC pre-treatment processes were able to remove most of the suspended and dissolved solids with removal efficiency ranging from 71 to 85%.

## 4. Conclusions

The performance of an IMF treatment system connected to an electrochemical (EC) process (pre-treatment) on the purification of a poultry slaughterhouse wastewater toward achieving a high-quality effluent has been studied. The analysis of the samples for physicochemical parameters was achieved at the ENU Water and Environmental Management lab in Kazakhstan. Operating cost analysis was also done based on the energy consumption of each unit.

From the analysis results, it was observed that the EC pre-treatment unit was highly effective on the removal of some physicochemical parameters such as turbidity, color, TSS, total iron, aluminum, COD, and BOD; with removal efficiency ranging from 71 to 85%. The performance was also reflected in the final effluent after the IMF treatment processes. The low removal efficiency of the pre-treatment system was also observed from free and total chlorine, nitrites, nitrates, phosphates, and total phosphorus, and ammonium nitrogen; with removal efficiency ranging from 4 to 45%.

In general, the treatment approach was observed to be highly efficient for some physicochemical parameters such as turbidity, color, TSS, COD, and BOD; maintaining almost 100% removal efficiency throughout the study period. Moreover, being supported by the EC pre-treatment processes, a low rate of cake formation was observed from the membrane filters.

Based on the results, we can conclude that the membrane filtration wastewater treatment technologies are relatively effective in terms of removal efficiency and operating cost for poultry slaughterhouse wastewater. However, apart from the characteristics of the poultry slaughterhouse wastewater, the performance of a membrane filtration system relies heavily on the type of pre-treatment technology used. The studied treatment system has the potential to be applicable in other types of wastewater, although sufficient pre-experiments may be necessary to optimize the system depending on the characteristics of the wastewater.

## Figures and Tables

**Table 1:** General characteristics of raw wastewater (number of samples = 12).

Indicators	Min	Max	AM	SD	Unit
pH	6.80	7.40	7.13	0.25	unitless
Turbidity	68.70	647.00	259.62	198.10	FAU
Color	552.00	8019.00	3147.83	2498.67	degree
TSS	116.00	1068.00	452.67	320.34	mg/L
Free chlorine	0	0.90	0.23	0.37	mg/L
Total chlorine	0.03	1.04	0.28	0.34	mg/L
Nitrites	0.11	0.19	0.16	0.03	mg/L
Nitrates	0.20	27.50	17.01	9.79	mg/L
Phosphates	2.67	5.16	3.82	0.93	mg/L
Ammonium	9.03	34.20	13.50	9.26	mg/L
Total iron	0.24	1.33	0.90	0.45	mg/L
Aluminum	0.66	0.89	0.78	0.12	mg/L
COD	1274.00	7401.00	3527.17	2125.54	mg/L
BOD	1220.00	1409.00	1321.33	69.20	mg/L
Chromium	0.56	2.10	1.19	0.64	mg/L
Nickel	1.96	21.96	7.53	6.88	mg/L
Manganese	0.29	0.95	0.72	0.22	mg/L

FAU = Formazin attenuation units; mg = milligrams; L = liter.

**Table 2:** The general setting of the treatment plant.

Performance	Unit	Value
Wastewater	m^3^/h	1.25
Treated water	m^3^/h	0.75
Drainage	m^3^/h	0.5
Water temperature	°C	+5–+40
Total power t	kW	3.8

**Table 3:** Interpretation of the correlation coefficients.

Range of Correlation Coefficient	Strength of Relationship
0–0.29	Weak
0.3–0.49	Moderate
0.5–0.69	Strong
0.7–1	Very strong

**Table 4:** Physicochemical analysis results (electrochemical (EC) phase effluent).

Indicators	Min	Max	AM	SD	Unit
pH	7.40	7.70	7.60	0.12	
Turbidity	1.08	114	70.32	41.92	FAU
Color	90	1239	802.75	429.11	degree
TSS	13	198	128.25	69.25	mg/L
Free chlorine	0	0.22	0.15	0.07	mg/L
Total chlorine	0	0.24	0.18	0.08	mg/L
Nitrites	0.06	0.21	0.14	0.05	mg/L
Nitrates	1.10	15.20	11.38	5.95	mg/L
Phosphates	1.08	4.59	3.66	1.49	mg/L
Ammonium	0.77	32.50	8.91	13.63	mg/L
Total iron	0.04	0.31	0.17	0.09	mg/L
Aluminum	0.20	0.26	0.22	0.02	mg/L
COD	70.60	1192	763.65	417.98	mg/L
BOD	48	448	198	159.34	mg/L
Chromium	0.24	1.96	0.72	0.72	mg/L
Nickel	2.20	7.20	3.61	2.08	mg/L
Manganese	0.12	0.42	0.21	0.12	mg/L

**Table 5:** Physicochemical analysis results (integrated membrane filtration (IMF) effluent).

Indicators	Min	Max	AM	SD	Unit
pH	7.70	8.14	7.95	0.19	
Turbidity	0	0.68	0.32	0.32	FAU
Color	5	19	8.83	5.37	degree
TSS	0	5	1.33	1.80	mg/L
Free chlorine	0	0.04	0.02	0.02	mg/L
Total chlorine	0	0.04	0.02	0.01	mg/L
Nitrites	0	0.01	0	0	mg/L
Nitrates	0.10	3.60	1.18	1.18	mg/L
Phosphates	0.02	0.48	0.12	0.16	mg/L
Ammonium	0	0.88	0.18	0.31	mg/L
Total iron	0.03	0.16	0.07	0.04	mg/L
Aluminum	0.02	0.21	0.05	0.07	mg/L
COD	1.12	31.20	8.46	10.25	mg/L
BOD	2.30	5.50	3.97	1.18	mg/L
Chromium	0.00	0.33	0.07	0.12	mg/L
Nickel	0.00	0.03	0.02	0.01	mg/L
Manganese	0.01	0.09	0.03	0.03	mg/L

**Table 6:** Summary of the estimated removal contributions.

Indicators	EC %	IMF %	Total RE %
Turbidity	72.91	26.96	99.88
Color	74.50	25.22	99.72
TSS	71.67	28.04	99.71
Free chlorine	21.17	69.34	90.51
Total chlorine	44.88	49.10	93.98
Nitrites	11.35	85.72	97.07
Nitrates	33.11	59.93	93.04
Phosphates	4.17	92.60	96.77
Ammonium nitrogen	4.43	93.61	98.03
Total iron	81.37	11.32	92.69
Aluminum	71.61	21.40	93.01
COD	78.35	21.41	99.76
BOD	85.02	14.68	99.70
Chromium	40.00	54.12	94.12
Nickel	52.03	47.7	99.73
Manganese	70.81	25.02	95.83

RE = removal efficiency.

**Table 7:** Compliance with drinking water quality standards.

Indicators	Average Concentration	Standard	% Compliance	Unit
pH	7.95	6.5-8.5	100	
Turbidity	0.32	5	93.60	FAU
Color	8.83	5	-76.60	degree
TSS	1.33	500 (KZ)	99.73	mg/L
Free chlorine	0.02	1.50	98.67	mg/L
Total chlorine	0.02	1.20 (KZ)	98.33	mg/L
Nitrites	0	3	100	mg/L
Nitrates	1.18	50	97.64	mg/L
Phosphates	0.12	3.50 (KZ)	96.57	mg/L
Ammonium	0.18	0.50 (KZ)	64	mg/L
Total iron	0.07	0.10	30	mg/L
Aluminum	0.05	0.10	50	mg/L
Chromium	0.07	0.05	-43.33	mg/L
Nickel	0.02	0.07	77.76	mg/L
Manganese	0.03	0.05	31.67	mg/L

KZ = water quality standards set by the government of Kazakhstan.

## Supplementary Materials

The following are available online at https://www.mdpi.com/2077-0375/10/10/256/s1. Table S1: Technical specifications of the pre-treatment units. Table S2: Technical specifications of the ultrafiltration unit. Table S3: Reverse osmosis technical specifications. Table S4: Correlation coefficients for some of the studied physicochemical parameters.
