# Supplementary material for: Performance of an Integrated Membrane Process with Electrochemical Pre-Treatment on Poultry Slaughterhouse Wastewater Purification
Source: Membranes (Basel). 2020 Sep 24;10(10):256. doi: 10.3390/membranes10100256 (PMC7599518; doi:10.3390/membranes10100256)
Supplement: Supplementary file 1 [file membranes-10-00256-s001.pdf]

Table S1. Technical specifications of the pre-treatment units.

Performance	Unit	Value
Feather catcher		
Length	mm	1040
Width		790
Height		1540
Fat catcher		
Length	mm	1000
Width		540
Height		1040
Mechanical filter for coarse cleaning		
Pressure	Bar	16
Nominal filtration fineness	µm	1000
Electrolysis		
Length	mm	1030
Width		820
Height		2030
Cathode		Graphite
Anode		Aluminium
Supply voltage	V	380
Hydraulic retention time (HRT)	min	24

Table S2. Technical specifications of the ultrafiltration unit

Performance	Unit	Value
Filter pore size	µm (kDa)	0.02 (1760)
Permeate	l/h	1060
Pressure	Bar	25
Pump supply voltage	V	220
Pump power	kW	0.44-0.7
Height	mm	1970
Area	mm	2100×850

Table S3. Reverse osmosis technical specifications

Parameter	Unit	Quantity
Permeate	l/h	1000
Temperature	^{0}C	15
Salinity	ppm	1500
Pressure	Bar	55
Power	kW	1.5
Voltage	U/Hz	380/50

	Height	mm	1933
Installation Size	Area	mm	2000×800

Table S4. Correlation coefficients for some of the studied physicochemical parameters

	**Turbidity**	**Color**	**TSS**	**Nitrates**	**Phosphates**	**Ammonium**	**COD**
Turbidity	1						
Color	0.999834	1					
TSS	0.999689	0.999978	1				
Nitrates	0.925642	0.932383	0.934785	1			
Phosphates	0.933783	0.940147	0.942411	0.999756	1		
Ammonium	0.581799	0.566883	0.561348	0.230771	0.25223	1	
COD	0.999923	0.999983	0.999921	0.930255	0.938141	0.571686	1
